# Exosome Determinants of Physiological Aging and Age-Related Neurodegenerative Diseases

**DOI:** 10.3389/fnagi.2019.00232

**Published:** 2019-08-28

**Authors:** Marianna D’Anca, Chiara Fenoglio, Maria Serpente, Beatrice Arosio, Matteo Cesari, Elio Angelo Scarpini, Daniela Galimberti

**Affiliations:** ^1^Department of Pathophysiology and Transplantation, Dino Ferrari Center, Faculty of Medicine and Surgery, University of Milan, Milan, Italy; ^2^Department of Clinical Sciences and Community Health, Faculty of Medicine and Surgery, University of Milan, Milan, Italy; ^3^Geriatrics Unit, Fondazione IRCCS Ca’ Granda, Ospedale Maggiore Policlinico, Milan, Italy; ^4^Neurodegenerative Diseases Unit, Fondazione IRCCS Ca’ Granda, Ospedale Maggiore Policlinico, Milan, Italy; ^5^Department of Biomedical, Surgical and Dental Sciences, Dino Ferrari Center, Faculty of Medicine and Surgery, University of Milan, Milan, Italy

**Keywords:** exosomes, aging, non-coding RNA, Alzheimer’s disease, frontotemporal dementia, Parkinson’s disease

## Abstract

Aging is consistently reported as the most important independent risk factor for neurodegenerative diseases. As life expectancy has significantly increased during the last decades, neurodegenerative diseases became one of the most critical public health problem in our society. The most investigated neurodegenerative diseases during aging are Alzheimer disease (AD), Frontotemporal Dementia (FTD) and Parkinson disease (PD). The search for biomarkers has been focused so far on cerebrospinal fluid (CSF) and blood. Recently, exosomes emerged as novel biological source with increasing interest for age-related neurodegenerative disease biomarkers. Exosomes are tiny Extracellular vesicles (EVs; 30–100 nm in size) released by all cell types which originate from the endosomal compartment. They constitute important vesicles for the release and transfer of multiple (signaling, toxic, and regulatory) molecules among cells. Initially considered with merely waste disposal function, instead exosomes have been recently recognized as fundamental mediators of intercellular communication. They can move from the site of release by diffusion and be retrieved in several body fluids, where they may dynamically reflect pathological changes of cells present in inaccessible sites such as the brain. Multiple evidence has implicated exosomes in age-associated neurodegenerative processes, which lead to cognitive impairment in later life. Critically, consolidated evidence indicates that pathological protein aggregates, including Aβ, tau, and α-synuclein are released from brain cells in association with exosomes. Importantly, exosomes act as vehicles between cells not only of proteins but also of nucleic acids [DNA, mRNA transcripts, miRNA, and non-coding RNAs (ncRNAs)] thus potentially influencing gene expression in target cells. In this framework, exosomes could contribute to elucidate the molecular mechanisms underneath neurodegenerative diseases and could represent a promising source of biomarkers. Despite the involvement of exosomes in age-associated neurodegeneration, the study of exosomes and their genetic cargo in physiological aging and in neurodegenerative diseases is still in its infancy. Here, we review, the current knowledge on protein and ncRNAs cargo of exosomes in normal aging and in age-related neurodegenerative diseases.

## Introduction

The growing increase of lifespan has implemented the research in aging processes and in age related pathologies like Alzheimer’s Disease (AD), Frontotemporal Dementia (FTD) and Parkinson’s disease (PD). Aging encloses multiple and complex processes where cellular senescence is the critical one. Senescent phenotype is characterized by three phenomena: the permanent cell growth arrest; the resistance to apoptosis; the acquisition of altered and differentiated functions (Campisi and d’Adda Di Fagagna, 2007; Campisi, 2012). Several evidence associates senescence to an increase in exosome release introducing a new phenotype named Senescence-Associated Secretory Phenotype (SASP) observed *in vitro* after genotoxic stress in different kinds of cells (Lehmann et al., 2008; Takasugi et al., 2017). Exosomes are tiny Extracellular vesicles (EVs) sizing from 30 nm to 100 nm, shed from almost all the cells, including the nervous ones (Zhang and Yang, 2018). Exosomes were thought to serve as cellular garbage but now there are many evidence that support their role in the intercellular communication (Rashed et al., 2017) pouring their content, through different mechanisms, to the recipient cells in the neighborhood as well as in the periphery even passing through the blood brain barrier (BBB; Alvarez-Erviti et al., 2011; Ridder et al., 2014). Indeed exosomes were detected in many biological fluids as in serum, plasma, urine, cerebrospinal fluid (CSF) and others (Caby et al., 2005; Franzen et al., 2015; Yagi et al., 2016). The growing interest in the last decade on exosome research is linked to their composition that represents a “mirror” of the physiological as well as the pathological state of the donor cells (Willms et al., 2016). Exosome cargo consists of lipid, proteins, mRNAs and ncRNAs, mostly microRNAs, whose sorting is regulated from the cell of origin with complex mechanisms that are not fully understood (Simons and Raposo, 2009). Instead, it is not clear, if the recipient cell can have an active role to select the exosome cargo or if it depends only from the parental cell. Not only their content but also markers on their membrane surface reflect their origin. Therefore, besides general exosome markers useful to discriminate exosomes from other EVs (e.g., CD81, CD9, ALIX, TSG101), the detection of neural derived exosomes (NDEs) is possible due to the presence of L1CAM (L1- cell adhesion molecule), that is a Central Nervous System (CNS)- specific exosome marker (Kenwrick, 2002; Fauré et al., 2006; Lachenal et al., 2010). Therefore, the investigation of the impressive variety of NDEs cargoes, especially proteins and microRNAs, could open a “window into the brain” creating a direct thread between the CNS and the periphery (Shi et al., 2019). To support this assumption, more and more findings have reported the presence of proteins and microRNAs critical for age-related disorders inside NDEs (Rajendran et al., 2014; Soria et al., 2017). This review article, is intended to explore the current understanding on the exosome’s role in physiological aging comparing to pathological aging in the most relevant elderly neurological disorders, such as AD, FTD and PD emphasizing the emerging discoveries on proteins and ncRNAs inside exosomes.

## Extracellular Vesicles (EVs)

**Figure 1:**
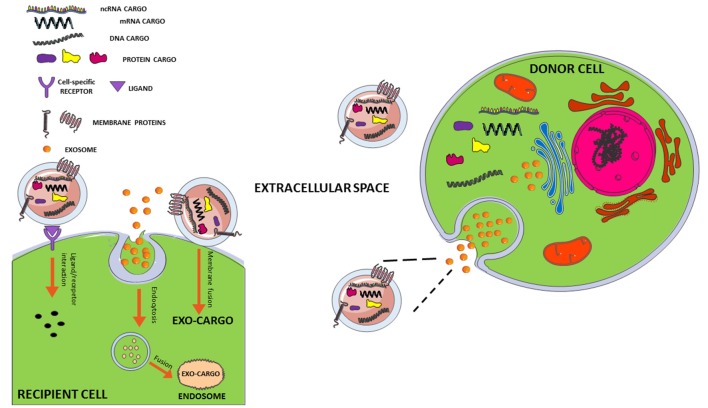
Interaction mechanisms between exosome and recipient cell. Exosomes secreted from a donor cell can move through biological fluids to reach the recipient cell close or distant to the site of their origin. The interaction mechanisms with recipient cell are various: (1) membrane fusion with transfer of exosomal cargo to recipient cell; (2) internalization of whole exosome by endocytosis and release of the cargo by fusion with endosomal membrane; (3) activation of signaling pathways through ligand/receptor interaction. For more details, see review by Jan et al. (2017, 2019).

**Figure 2:**
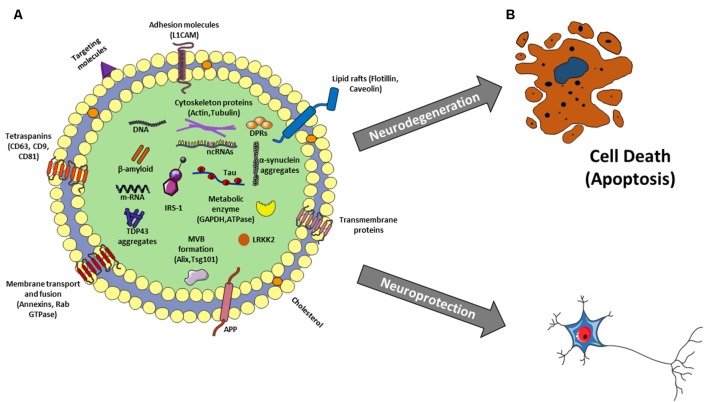
**(A)** Exosome structure and cargo. Exosomes are surrounded by a phospholipid bilayer and their content reflects the cell of origin. So besides to generic molecules that identify all exosomes as Tetraspanins, Lipid rafts, MVB formation proteins, membrane transport and fusion proteins, there are other molecules specific to their origin, as L1CAM for neural exosomes as well as proteins related to aging and neurological diseases such as β-amyloid, p-Tau, LRKK2, insulin receptor substrate 1 (IRS-1), a-synuclein, amyloid precursor protein (APP), TDP-43, depeptide protein repeats (DPRs) and many others. In addition, there is also a nucleic component consisting of DNA, mRNA and different kinds of non-coding RNAs (ncRNAs; miRNAs, lncRNAs, circRNAs, piwi-RNA, etc…). **(B)** Exosomes: a double-edged sword in neurodegenerative disease. Exosomes could favorite and/or trigger the spreading of the disease leading to neurodegeneration or they could sequester neuro-toxic components from neural cells protecting them.

EVs are membrane surrounded structures released outside the cells. To date, these vesicles have been cataloged-based on their dimension and origin. Among those exosomes, originating from the endosomal compartment, are the most investigated. They have small dimensions (30–100 nm) and round shape (Mashouri et al., 2019). The biochemical content of exosomes consists of lipid, proteins but also microRNA and mRNAs. Several studies reported that mRNAs delivered by exosomes to target cells were translated in functional proteins (Pegtel et al., 2010); in the same way miRNAs regulated gene expression in recipient cells (Figure 1; Hu et al., 2019). Moreover, it has been reported the presence of genomic and mitochondrial DNA (Hough et al., 2018). Exosome contents not only reflect the donor cell composition but also reflect a sophisticated sorting mechanism. Analysis of exosome proteome revealed that some proteins specifically arise from cell and tissue of origin, and some are characterisitic for all exosomes (Figure 2A; Mashouri et al., 2019). The lipid content of exosomes is cell-specific or conserved. Indeed lipids protect exosome shape, take part in exosome biogenesis, and regulate homeostasis in the recipient cells (Vidal et al., 1989). Noteworthy, exosomes are present in several body fluids such as blood, urine, breast milk, saliva and also CSF (Urbanelli et al., 2016). Given that, they appear potentially useful biomarkers for the diagnosis of several diseases, including neurodegenerative diseases.

### Exosomes in Aging and Cellular Senescence

The increasing number of aged individuals in the global population will likely lead to an increase of costs accounting on health care system. Thus, it is of crucial importance improving the comprehension of the mechanisms underneath ageing processes and developing new therapeutic strategies in order to reduce the effects of age-related morbidities. The goal standard will promote an improvement in health lifespan and a reduction of age-related co-morbidities conditions.

Aging is defined as a loss of physiological function during the time and is regulated by specific molecular pathways (Xu and Sun, 2015). Aging process leads to an increased risk of several chronic diseases such as cancer, cardiovascular disease, and autoimmune disease, but also dementia. It is associated with the body’s altered capacity to face up stress caused by metabolism, infection, and damage to cellular macromolecules. The comprehension of molecular mechanisms driving aging will help the scientific community to figure out why aged individuals are more vulnerable to those diseases and why they may be less stress-resistant (Panagiotou et al., 2018).

Aging is considered conserved across *taxia* and it is characterized by nine hallmarks comprising: genomic instability, telomere shortening, epigenetic alterations, loss of proteostasis, deregulated nutrient sensing, mitochondrial dysfunction, senescence, stem cell exhaustion and alteration in intercellular communication (Shiels et al., 2017). Moreover, the so-called “inflammaging,” a chronic inflammatory status, represents the main feature of aging process (Salvioli et al., 2013).

While aging involves the entire organism, not all cell types age at the same rate and it is conceivable that senescent cells may contribute to spread senescence to young cells (Olivieri et al., 2015).

Senescence is a particular phenotype of eukaryotic cells leading to a loss of replication ability in response to several stimuli that induce DNA damage (Campisi and d’Adda Di Fagagna, 2007).

The major component in the signal transmission from senescent cells to the surrounding tissue is the SASP that can facilitate the removal of senescent and remodeling of tissue by attraction of phagocytic immune cells (Urbanelli et al., 2016). Beside previously known SASP components, many Wnt ligands have been counted. Wnt is a secreted signaling molecule extremely conserved playing critical roles in many processes including stem cell proliferation and maintenance of homeostasis in the canonical pathway (β-catenin dependent) and transcriptional and non-transcriptional cellular responses in the non-canonical ones (β-catenin independent) triggered by calcium or other Wnt ligands, as Frizzled receptors (Nusse, 2005). It should be noted that these two different pathways interact with each other and other multiple pathways, including the NF-κB, MAPK, and JNK pathways making Wnt signaling extremely complex and articulated (Zhang et al., 2014; Ma and Hottiger, 2016). It is not surprising that Wnt is involved in aging too (Nusse, 2005). Indeed some sources suggest that Wnt signaling decays with aging in brain impairing adult neurogenesis (Okamoto et al., 2011) and lung (Hofmann et al., 2014 but at the same time it may increase in an age-dependent manner (Brack et al., 2007; Liu et al., 2014). Furthermore, the key members of Wnt pathways involved in SASP are secreted in the extracellular space by exosomes. These vesicles carrying Wnt proteins on their surface have been reported to active Wnt signaling in target cells. These findings highlighted a new role of exosomes in mediating the cell-to-cell transmission of senescence signals, suggesting that exosomes represent a new SASP component (Urbanelli et al., 2016). For the first time in 2008, Lehmann et al. (2008) described an increase of exosomes secretion by senescent cells. This increase seems to be a general feature of cellular senescence and has been observed in fibroblasts, epithelial cells, and cancer cells (Takasugi, 2018). On the contrary, Eitan et al. (2017) in cross-longitudinal study showed that plasma exosomes concentration decreased with human age, at least from the early 30s to late 60s. Monocytes and B cells internalized more exosomes rather than T cells, even if these ones are the most representative kind of PBMC in bloodstream. In addition, exosomes were more incorporated in B cells and monocytes of aged donors suggesting that exosomes internalization not only is cell-specific but age-dependent. Moreover, aging can alter RNA and protein composition of exosomes. For example Galectin-3, which plays a role in osteoblast maturation, was reduced in the plasma exosomes of elderly people, presumably as consequence of the stem cell functionality loss in the skeleton, classical of aging process (Weilner et al., 2016). Plasma exosomes isolated from young but not elderly donors promoted the osteogenic differentiation of mesenchymal stem cells in a galectin-3-dependent manner (Weilner et al., 2016). In particular, this protein, belonging to the lectin family, consists of carbohydrate recognition and collagen α-like domains. This chimeric structure allows Galectin-3 to interact with a multitude of intra-and extracellular proteins, in the nucleus as well as in the cytoplasm or on the membrane and in the extracellular space, after its secretion from different types of cells and tissues. Interacting with a myriad of proteins, Galectin-3 is involved in multiple biological processes, physiological and pathological, such as development, neuronal functions, immune reactions, endocytosis, neoplastic transformation and metastasis, and osteoblastogenesis, which impairing seems to contribute to age-related bone frailty (Dumic et al., 2006).

Exosomal miRNAs are also involved in brain aging (Pusic and Kraig, 2014). Peripheral exosomes isolated from young Wistar rats promoted differentiation in primary oligodendrocyte precursor cell (OPC) differentiation and remyelination in slice cultures. Moreover, nasal administration of EVs from young rats increased myelination in aged rat brain due to the presence of high levels of miR-219, which reduced the expression of inhibitory regulators of OPC differentiation (Pusic and Kraig, 2014). Recently, it has been described that the activity of acetylcholinesterase protein (AChE) was increased in young as well as old Wistar rats. An age-related increase was observed in CD63 levels in CSF exosomes but a decrease was observed in plasma vesicles of the older group. The authors showed that the young adult rats had significantly higher circulating IL-1β levels in the exosomes compared to the aged ones, without any effect on central content. These data suggest that the normal aging process caused different changes in the profiles of central and circulating exosomes. Altered IL-1β levels in circulating EVs could be linked, at least partly, to age-related inflammatory conditions, and a disruption of the CSF exosomes in aged rats, evaluated by CD63 levels, could be related to susceptibility to neurodegenerative disorders (Gomes de Andrade et al., 2018).

Cellular senescence triggered by specific conditions as irradiation, DNA-damaging reagents, and oncogenic RAS expression, all enhance exosomes secretion. This increase is mediated by p53 (Lehmann et al., 2008) and one of its targets, Tumor suppression-activated pathway 6 (TSAP6), but the mechanism whereby TSAP6 regulates exosomes secretion is not well understood. Nevertheless, it has been demonstrated that exosomes creating pro-inflammatory environment accelerate the aging process (Biran et al., 2017). Interestingly, exosomes contain various lengths of genomic DNA fragments and seem to be one of the major routes of DNA secretion (Fernando et al., 2017) and DNA secretion from exosomes increases upon cellular senescence (Takasugi, 2018). Intriguingly, cH2AX positive cytoplasmic chromatin fragments appear in senescent cultured cells (IMR90 and HEMa-LP); suggesting that damaged DNA may be the major source of exosomes associated DNA in senescent cells (Ivanov et al., 2013). Exosomes are involved also in promoting genomic instability, another aging hallmark, thought the transfer of retrotransposons that are DNA elements able to create and insert multiple copies of themselves into host genomes. It is interesting to note that retrotransposons expression has been found to increase in senescent mouse cells (strain C57BL/6; De Cecco et al., 2013).

The deposition of toxic proteins is another event correlated with aging. To date, it is demonstrated that exosomes are involved in the transport of pathogenic proteins in the brain and in the progression of neurodegenerative diseases (Bellingham et al., 2012). Recently, the NDE levels of six neuronal proteins have been quantified in cognitively intact older subjects. Except for Phosphorylated tau-S396, the exosomal levels of Phosphorylated tau P-181, Beta Amyloid 42 (Aβ1–42), chatepsin D, repressor element 1-silencing transcription factor (REST) and neurogranin are significantly modified with aging (Goetzl et al., 2016).

### The Role of Exosome miRNAs in Aging and Cellular Senescence

MicroRNAs are short non-coding RNAs that regulate negatively gene expression at post-transcriptional level. It has been reported that several miRNAs are involved in aging and cellular senescence (Urbanelli et al., 2016). The importance of exosomal miRNAs analysis lies in the fact that these molecules could potentially transmit signals to surrounding tissues with a good impact, but also with a detrimental role. Moreover, exosomal miRNAs are interesting in the context of aging biomarker search (Sprott, 2010). MiRNAs released from senescence cells in the extracellular environment by exosomes have been reported that are able to spread senescence in surrounding cells.

For example, miR-433 promoted the induction of senescence in ovarian cancer cells (A2780) and when overexpressed, miR-433 was released in association with exosomes (Weiner-Gorzel et al., 2015). MiR-34a and miR-29 can induce cell cycle arrest in colon carcinoma cell line (HCT116 cells) contributing to the stabilization of p53/p21 by targeting proteins relevant for its regulation such as Sirtuin 1 (SIRT1; Yamakuchi and Lowenstein, 2009). The miR17–92 cluster is down regulated in several cell aging models as endothelial cells, replicated CD8+ T cells, renal proximal tubular epithelial cells, and skin fibroblasts and they can target p53/p21 (Weilner et al., 2013). Another miRNA involved in cellular senescence is miR-146, whose expression was increased in senescent human fibroblasts (HCA2) when compared with proliferating quiescent ones. It is important to underlie that miRNA-146 targets IL-6 and IL-8, SASP components with pro-inflammatory function, suggesting a role for miR-146 as senescence-associated inflammation modulator (Bhaumik et al., 2009). On the other hand, miRNAs encapsulated in exosomes are able to suppress cellular senescence; this is the case of miR-214, involved in angiogenesis, that plays a role in vesicle-mediated signaling between endothelial cells. Exosomes derived from human microvascular endothelial cell line (HMEC-1) stimulated migration and angiogenesis in recipient cells, whereas exosomes from miR-214-depleted endothelial cells failed to stimulate these processes preventing senescence and allowing blood vessel formation (van Balkom et al., 2013). A recent microarray study performed on salivary exosomes miRNAs from young and old healthy subjects has identified mir-24-3p as a possible peripheral aging biomarker (Machida et al., 2015). Exosomes isolated from the bone marrow of young and aged C57BL/6 mice showed a similar concentration and size distribution. However, bioanalyzer data indicated that exosomes from young and aged mice were differently enriched in miRNAs. The amount of miR-183-5p was increased in aged bone marrow exosomes, and its overexpression detected also in bone marrow stromal cells, mimicked the effects of aged bone marrow exosomes (Davis et al., 2017).

## Exosomes in Elderly Neurological Disorders: Neuroprotective or Neurodegenerative Role?

Although there are, still few evidence on the role of exosome in the healthy aged brain, as we discussed before, it is known that exosomes have a role in the pathogenesis and in the progression of many neurodegenerative diseases (Soria et al., 2017). However, it is not established if they play a positive or negative role because the literature is controversial defining them like a double-edged sword in the neurodegenerative disease (Lee and Kim, 2017). The discovery that exosomes carry functional biomolecules as key pathogenic proteins (e.g., Aβ-amyloid, tau and α-synuclein) and miRNAs (Figure 2A) led to consider their involvement in neurological disorders (Thompson et al., 2016). Dysregulation of intercommunication between neurons or between neurons and glial cells mediated by exosomes could trigger the disease (Lee and Kim, 2017). On the contrary, exosomes could sequester neuro-toxic components from neural cells and flow neuro-protective ones (Figure 2B). This means that they can favorite the spreading of the disease or they can inhibit it (Lee and Kim, 2017). This section is intended to give an overview of the double roles of exosome proteins and microRNAs proposed for AD, FTD and PD.

### Exosomes in Alzheimer’s Disease (AD)

**Figure 3:**
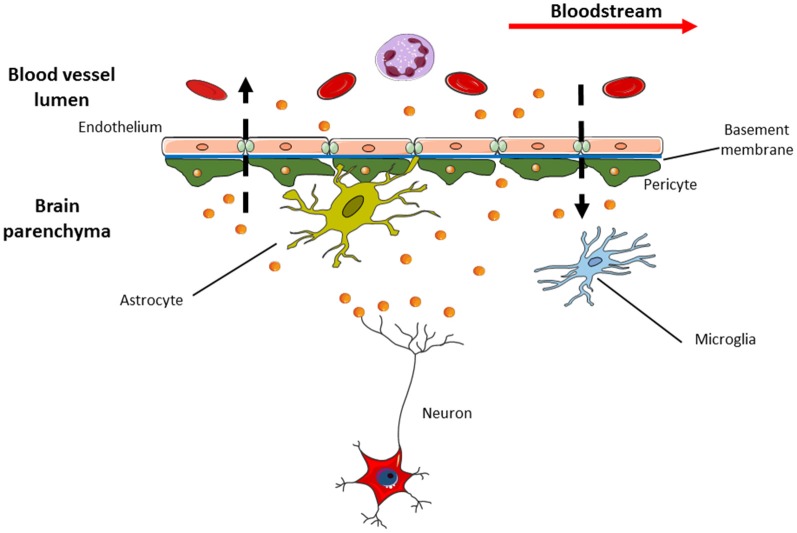
Exosomes are able to pass through blood brain barrier (BBB) in both directions. This means that specific exosomes (i.e., the neural ones with L1CAM on their surface) detected in the cerebrospinal fluid (CSF) can be released into bloodstream and vice versa. This feature makes exosomes appealing in the research of new sources of biomarkers suitable for use in clinical practice, as liquid biopsy that could replace current invasive diagnostic methods.

AD is considered the most frequent cause of dementia. It is characterized, clinically, by cognitive and behavioral disorders and, pathologically, by the extracellular deposit of insoluble Aβ-amyloid and intracellular neurofibrillary tangles (NFTs), consisting of tau fibrils. The amyloid plaques derived from impaired processing of the APP leading to the formation of the toxic Aβ-amyloid. APP is translocated into the endoplasmic reticulum (ER) and matures through Golgi apparatus. The mature form of APP is transported to the cell membrane where it undergoes to further proteolytic cleavage from β- and γ-secretases acting together to produce fibrils of the toxic Aβ-amyloid that accumulates with age in human AD brains (Busciglio et al., 1993; Takahashi et al., 2002). The dosage of Aβ-amyloid, total tau and phosphorylated tau in the CSF is recognized as the “core” of AD biomarkers in the clinical practice (Molinuevo et al., 2018). Interestingly, the first link between exosomes and AD proposed that Aβ-amyloid was released in association with exosomes. Moreover, the presence of other specific exosomal proteins as Alix and Flotillin-1 were also found accumulating into the AD brain (Rajendran et al., 2006; Sharples et al., 2008). A prion-like mechanism to explain how aggregates of Aβ-amyloid seem to self-propagate and spread to cells out of CNS is confirmed in AD mouse models. Seeding of Aβ was observed when extracts of AD human brain were injected in healthy mice that express the human wild-type APP gene causing the formation of the plaques in the site of injection and adjacent brain region (Morales et al., 2011). In addition, tauopathy was inducted in ALZ17 transgenic mice injecting aggregated of tau protein (Clavaguera et al., 2009). In the light of these findings, the hypothesis that exosomes could use a prion-like mechanism to disseminate toxic proteins associated with AD is taking hold (Coleman and Hill, 2015; Thompson et al., 2016). Exosomes could be involved in the trafficking of amyloid aggregates because Tg2576 mouse brain mice and post-mortem human AD brains were enriched in exosome markers within amyloid plaques (Kokubo et al., 2004; Rajendran et al., 2006). Phosphorylated tau was also detected in the exosomes from CSF of early-onset AD patients (Saman et al., 2012). ADAM10, Beta-secretase 1 (BACE1), nicastrin, and presenilin 1 and 2 (PSEN1 and 2) are other examples of AD pathogenic proteins found inside exosomes of transgenic mouse brain (Tg2576) and cell culture APP models, as CHO cell line (Sharples et al., 2008; Perez-Gonzalez et al., 2012). More recently, researchers have found that exosomes could stimulate aggregation of Aβ-amyloid and tau *in vivo* models, 5XFAD and rTg4510 transgenic mice (Dinkins et al., 2014; Polanco et al., 2016). In other words, exosomes, removing the excess of intracellular Aβ, shuttled it outside the cells concurring to plaque formation (Joshi et al., 2015). On the other hand, a neuroprotective role is also proposed. Neural exosomes could uptake Aβ-amyloid reducing the Aβ load in the brain as seen in the brains of mouse models (C57BL/6, KM670/671NL and V717F) where after the injection of exosomes, a decrease of Aβ and amyloid deposition was observed (Yuyama et al., 2015). Furthermore, extracellular tau could arise by secretion through exosomes in SH-SY5Y and COS-7 cell lines (Simón et al., 2012). Even if there is a body of literature arguing the role of exosomes in aggregate transmission, the fact remains that this theory assumes the presence of pathogenic proteins within exosomes about which the functional evidences are few or controversial (Lim and Lee, 2017). Nevertheless, the exosome hypothesis is appealing and partly explains the intercellular transmission of proteinopathies. Worth mentioning also, a research field that proposes exosomes as source of biomarkers for CNS disorders (Figure 3) due to their interesting characteristics suitable to the clinic (e.g., presence in many biological fluids, crossing the BBB, protection of the biomolecules inside them, etc.). Goetzl et al. (2015, 2018) measured the levels of different pathogenic proteins, Aβ-amyloid, total tau and p-tau isoforms inside NDEs immunoprecipitated with L1CAM to isolate specifically neuronal exosomes from blood of AD, Mild Cognitive Impairment (MCI) and controls (Kapogiannis et al., 2015). They found higher levels of these proteins vs. controls able to predict the development of AD 10 years before clinical onset (Fiandaca et al., 2015) or the progression from MCI to dementia (Winston et al., 2016). Instead, a contrary study showed no difference in NDEs total tau levels for AD patients (Shi et al., 2017). It is known that type-2 diabetes is an AD risk factor thus AD brains have markers of insulin resistance as Insulin Receptor Substrate-1 (IRS-1). Altered forms of IRS-1 were detected in NDEs of AD plasma patients and at lower levels compared to controls and to patients with type 2 diabetes with intermediate levels. This is interesting because NDEs IRS-1 protein levels could contribute to discriminate MCI/AD to controls and patients with type-2 diabetes at the same time (Kapogiannis et al., 2015). Pathological proteins were also found in exosomes extracted from CSF. In the work of Saman et al. (2012), the tau phosphorylated at threonine 181 (pT181) was more concentrated in CSF exosomes than in the total CSF and in early stage of AD, while it was absent in other dementing conditions as vascular or Lewy body diseases. This exosomal tau detected so early in AD suggests that CSF tau could be secreted, not shed from dead neurons (Saman et al., 2012).

#### The Role of Exosome miRNAs in AD

Not only proteins but also ncRNAs, mostly miRNAs, are detected within exosomes and are different from those of donor cell. These so named “exosomal RNAs” are shuttled between donor and recipient cells becoming “exosomal shuttle RNA” (esRNA). They are protected from the degradation and are functionally active suggesting the esRNAs as a novel mechanism of intercellular genetic transfer and communication (Valadi et al., 2007). Exosomal miRNAs have been isolated from exosomes derived from different kinds of cells (from C57BL6 primary cultures) including neurons and primary astrocytes (prepared using cortices obtained from neonatal rat) and fluids as blood and CSF (Caby et al., 2005; Guescini et al., 2010; Goldie et al., 2014; Liu et al., 2014; Cheng et al., 2015; Lugli et al., 2015). The relevance of miRNAs in the CNS is now widely documented with almost 70% of all miRNAs expressed in the human brain (Nowak and Michlewski, 2013) hypothesizing that neuronal miRNAs may regulate the transcription of more than a third of genes (Kosik, 2006). Therefore, it is not surprising that altered blood/CSF exosomal miRNAs signature could be related to neurodegenerative disease, in particular to AD (Cheng et al., 2015; Gui et al., 2015; Lugli et al., 2015). Cheng et al. (2015) profiled miRNAs from serum exosomes to determine a set of miRNAs differentially expressed in AD. They found a specific miRNAs signature consisting of 16 miRNAs, along with risk factors, and many of them were identified as implicated in AD pathogenesis in several mouse and cell models. In detail, mir-1306-5p, that targets ADAM10, was the microRNA with the best sensitivity and specificity to predict AD. Lugli et al. (2015) found another interesting microRNA signature in plasma exosomes using Illumina deep sequencing technology. The researchers identified 20 microRNAs downregulated among which the lowest expressed miRNA in AD group compared to controls was the miR-342-3p. This is a brain-enriched miRNAs and its expression was highly correlated across individuals. Interestingly, the failure of proteasomal machine in tauopathies was supposed to be modulated by miRNA expression (Carrettiero et al., 2009). Indeed, hyperphosphorylation of tau was linked to up-regulation of ERK kinases after downregulation in AD brains of mir-15a, specifically dysregulated in AD (Hébert et al., 2010).

**Figure 4:**
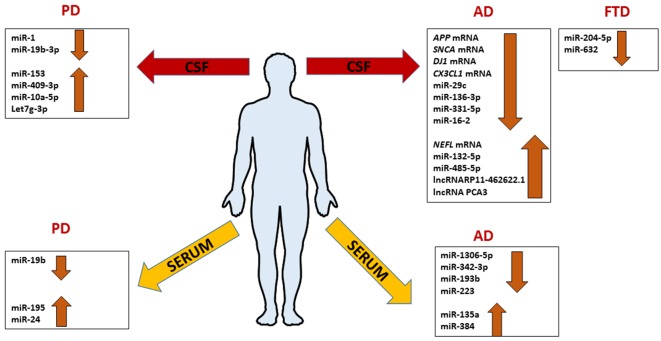
Comprehensive expression profile patterns of ncRNAs differentially expressed in patients with Alzheimer’s disease (AD), frontotemporal dementia (FTD) and Parkinson’s disease (PD) extracted from serum or CSF exosomes.

AD-related exosomal microRNAs were also investigated in the CSF. The overexpression of mir-193b in the hippocampus of AD C57BL/6J double transgenic mice could inhibit the expression of APP involving it in neurodegenerative process like an unique biomarker of AD (Liu et al., 2014). Gui et al. (2015) performed another study on exosomes from CSF. They isolated exosomes in CSF from AD patients and healthy controls, and used microarray analysis in order to identify microRNAs differentially abundant between AD, and normal group. AD exosomes showed fewer differences with healthy controls, with only six miRNAs showing significantly altered levels. In the same study, it was interesting to notice that also several mRNAs were differentially expressed in CSF exosomes in AD subjects. The levels of *APP* mRNA, *SNCA* (α-synuclein) mRNA, *DJ-1/PARK7* (Deglicase) mRNA, and *CX3CL1* (Fractalkine) mRNA were lower in AD exosomes, while the levels of neurofilamentL (*NEFL*) mRNA were higher. Interestingly, *MAPT* (Tau) mRNA was unchanged while the lncRNAs RP11- 462G22.1 and PCA3 were enriched in CSF exosomes from AD (Figure 4; Gui et al., 2015).

The potential therapeutic utility of exosomes is nowadays increasing. For example, siRNAs inside exosomes could use to target specific genes. Alvarez-Erviti et al. (2011) demonstrated that exosomes with exogenous siRNA anti-BACE1 were able to reduce the levels of BACE1 mRNA and protein in C57BL/6 mouse model brains. As well as the number of researches proposing one or more exosomal miRNAs as a potential biomarker to prognostic and/or diagnostic AD is growing. Recently, Yang et al. (2018) reported that serum exosome miR-135a and miR-384 were up-regulated while miR-193b was down-regulated in the serum of AD patients compared with normal controls, whereas exosomal miR-384 was the best among the three miRNAs to discriminate AD, Vascular Dementia (VaD), and PD with dementia (PDD). Receiver Operating Characteristic (ROC) curve to estimate the diagnostic utility of a biomarker or a set of them showed that the combination of miR-135a, -193b, and -384 was better than the single one to diagnose early-onset AD (Yang et al., 2018). Another study analyzed a limited subset of miRNAs involved in neuroinflammation, miR-137, miR-155 and miR-223 (Figure 4). They found that the median level of serum exosomal miR-223 was significantly reduced in patients with AD and was significantly correlated with Mini-Mental State Examination (MMSE) scores, Clinical Dementia Rating (CDR) scores, magnetic resonance spectroscopy (MRS) spectral ratios and serum concentrations of IL-1b, IL-6, TNF-a, and CRP. Authors concluded that exosomal miR-223 could be a promising biomarker for AD diagnosis although the sample size was limited and miRNAs screened are only three (Wei et al., 2018). Although both works are certainly interesting, they should be considered with attention because they lack the correlation with CSF values of β-amyloid, tau and P-tau. Anyway, they are pioneering for future studies.

It should be mentioned that exosomes have inside them other categories of ncRNA species as long noncoding RNAs (lncRNAs), circular RNAs (circRNAs), small nucleolar RNA (snoRNAs), small nuclear RNAs (snRNAs), transfer RNA (tRNAs), ribosomal RNAs (rRNAs), and piwi-interacting RNAs (piRNAs) identified comprehensively using high-throughput RNA-Seq (Kim et al., 2017). Although the role for some of them is emerging as critical for gene expression, their involvment in AD related to exosomes is still in the infancy.

### Exosomes in Frontotemporal Dementia (FTD)

FTD is the most common form of dementia in the presenium accounting for up to 20% of patients with an onset before 65 years. Three clinically different syndromes characterize FTD: behavioral variant (bv) FTD, Progressive Non Fluent Aphasia (PNFA), and Semantic Dementia (SD). These different subtypes are related to different clinical features but mostly patients present a profound alteration in the behavior and personality, often associated with cognitive and executive impairment, except for PNFA and SD where the language impairment is prevalent (Snowden et al., 2007). All of these syndromes at pathological level are characterized by Frontotemporal lobar degeneration (FTLD). Histopathologically FTLD is defined on the type of protein depositing into FTLD-Tau, FTLD-TAR DNA Binding protein (TDP)-43, and FTLD-Fused in Sarcoma (FUS; Fenoglio et al., 2018).

Up to 40% of patients have a history of familial transmission with nearly 10% of patients showing an autosomal dominant inheritance pattern. The majority of familial FTLD account mutations in the microtubule associated protein tau (*MAPT*) and progranulin (*GRN*) genes, and the pathologic expansion of the hexanucleotide GGGGCC repeat in the first intron of *C9ORF72* gene (Rademakers and Hutton, 2007).

As discussed above for AD, also for FTD, the involving of exosomes in the pathology has been investigated although the current knowledge is still limited. FTD is characterized by TDP-43 aggregates accumulation throughout the nervous system. As for AD pathogenic proteins, also TDP-43 protein can be exchanged *via* exosomes between neuronal cells (Neuro2a cells and primary neurons) leading to propagation of TDP-43 proteinopathy in a “prion-like” manner (Iguchi et al., 2016). Indeed the uptake of exosomal TDP-43 oligomers from recipient cells induces higher toxicity than free TDP-43 in murine primary cortical neuron cell culture (C57Bl76J; Feiler et al., 2015). Furthermore, exosomes derived from ALS-FTD-CSF cell model showed a high concentration of full length and TDP-43 C-terminal fragments (CTFs). The latter lead to the formation of cytoplasmic inclusions within cells, so authors suggest that aberrant cleavage of TDP-43 in these exosomes acting as “seed” induces the formation of TDP-43 aggregates in the ALS-FTD-CSF-cultured cells (Ding et al., 2015). Not only TDP-43, but also dipeptide repeat proteins (DPRs) produced by aberrant translation of *C9ORF72* FTD patients throughout CNS seem to spread between cells *via* exosome-dependent pathways (Westergard et al., 2016).

As was the case for AD patients, levels of Aβ and pT181 were increased in FTD as well (Fiandaca et al., 2015). Interestingly, the levels of synaptophysin, synaptopodin, synaptotagmin-2, and neurogranin dosed in NDEs were decreased in patients with FTD compared to controls, probably because of reduced functionality of synaptic proteins in senile dementias. These levels were low years before dementia making synaptic NDEs proteins useful for preclinical diagnosis of dementia (Goetzl et al., 2016). Instead, the Repressor Element 1 Silencing Transcriptor factor (REST) was significantly high in FTD over controls and AD representing a potential marker to discriminate FTD patients from AD (Goetzl et al., 2015). The IRS-1 phosphorylated in serine 312 was able to distinguish at 84% of accuracy between FTD patients and controls (Kapogiannis et al., 2015).

Lastly, Benussi et al. (2016) studied human primary fibroblasts without GRN null mutations. They conclude that the glycosylated form of PGRN was released with exosomes and in the presence of mutation, the secretion of exosomes was extremely reduced and their composition changed enriching in Lamp1 protein. Overall, the GRN null mutations cause an alteration in the intercellular communication.

#### The Role of Exosome miRNAs in FTD

The current knowledge of exosomal miRNAs in the pathogenesis of FTLD is exiguous. The work of Schneider et al. (2018) is the only performed on exosomes from CSF of FTLD patients. MiRNA expression profiles of 23 presymptomatic and 15 symptomatic mutation carriers compared to 11 healthy non-mutation carriers were performed on the Genetic Frontotemporal Dementia Initiative (GENFI) cohort and sporadic FTD. They found that miR-204-5p and miR-632 significantly decreased in symptomatic respect to presymptomatic mutation carriers (Figure 4). In another cohort, the miR-632 was highly decreased in sporadic FTLD compared to sporadic AD and healthy controls. The authors, using *in silico* analysis, discovered a potential target of miR-204-5p and miR-632; HRK that encodes for HARAKIRI, a pro-apoptotic protein. Its aberrant increasing could contribute to the neuronal death in FTLD patients (Schneider et al., 2018). Although these findings open a new perspective in the FTLD research, they need further investigations.

### Exosomes in Parkinson’s Disease (PD)

PD is a chronic neurodegenerative disease characterized by motor impairments due to the selective death of dopaminergic neurons. Cognitive impairments can arise in the course of the disease at any time. The most of PD cases are sporadic but there are rare familial forms linked to mutations in several genes; *SNCA, parkin, DJ-1*, PTEN-induced kinase 1 (PINK-1) and Leucine-rich repeat kinase 2 (LRRK2; Thomas and Beal, 2007). Even if the molecular pathogenesis of PD is not fully understood, it’s now universally accepted that α-synuclein plays a predominant role in PD accumulating in Lewy Bodies, a pathological hallmark of PD (Spillantini and Goedert, 2018). Indeed α-synuclein aggregates are responsible for synaptic pathology and neurodegeneration (Kramer and Schulz-Schaeffer, 2007). In addition, mutations that involve duplication or triplication of the wild-type *SNCA* are associated to autosomal dominant PD with a severity proportional to the degree of α-synuclein over-expression whereas missense mutations in *SNCA* (e.g., A53T) are linked to dominantly inherited forms of PD (Thomas and Beal, 2007). Therefore, it is not surprising that the spreading of pathology, already proposed for AD and demonstrated for PD involved α-synuclein. Several studies on murine primary cortical neurons and SH-SY5Y cell lines reported that α-synuclein was secreted from exosomes (Emmanouilidou et al., 2010; Danzer et al., 2012). Moreover, exosomes, providing environments for α-synuclein nucleation, catalyzed its aggregation in N2a cells and cultured hippocampal neurons (Olanow and Brundin, 2013; Grey et al., 2015). Another study in human H4 cell line demonstrated that the loss of function of P-type ATPase ion pump PARK9/ATP13A2 led to a decrease in secretion of α-synuclein into extracellular space, indeed the overexpression of PARK9/ATP13A2 caused the opposite effect, suggesting that PARK9/ATP13A2 was involved in the α-synuclein secretion at least in part *via* exosomes (Tsunemi et al., 2014). This consequence could have a neuroprotective effect. Probably because the increased release of exosomes containing α-synuclein reducing the intracellular levels of that protein, it could explain the surviving of neurons of substantia nigra in sporadic PD patients that overexpress PARK9/ATP13A2.

Furthermore, the biogenesis of α-synuclein exosomes seems to be modulated by zinc levels regulated from PARK9/ATP13A2 in SHSY5Y cells (Kong et al., 2014). Stuendl et al. (2016) measured the levels of CSF exosomal α-synuclein, and found differences among patients with PD and Lewy bodies. In accordance with previous studies in glioblastoma cell lines, the same group demonstrated that CSF exosomes derived from patients with PD and dementia with Lewy bodies induced the oligomerization of soluble α-synuclein in target cells in a dose-dependent manner (Stuendl et al., 2016). In this regard, Shi et al. (2014) were able to demonstrate in mouse models that CSF α-synuclein was promptly transported to blood, with a small portion within exosomes but CNS specific. An increased releasing of this protein to the blood of PD patients was explained discovering that in a large cohort of clinical samples (267 PD and 215 controls); the levels of plasma exosomal α-synuclein were significantly higher in PD patients. Another protein secreted from exosomes is LRRK2. Mutations in the *LRKK2* gene cause late-onset PD. LRKK2 secretion was regulated by 14-3-3 protein. Indeed, using 14-3-3 inhibitor, the LRRK2 secretion from exosomes was interrupted in mouse primary neurons and macrophages (Fraser et al., 2013).

#### The Role of Exosome miRNAs in PD

Concerning the involvement of exosomal miRNAs in PD, the literature is still scarce. Cao et al. (2017) profiled the expression of 24 candidate miRNAs, already dysregulated in previous studies, in the serum of 109 PD patients matched with healthy controls finding the downregulation of miR-19b and the upregulation of miR-195 and miR-24 compared to healthy controls. Instead, Gui et al. (2015) investigated the expression of 746 miRNAs in CSF of PD patients finding 16 miRNAs upregulated and 11 downregulated. In detail, miR-1 and miR-19b-3p were significantly reduced; miR-153, miR-409-3p, miR-10a-5p, and let-7g-3p were significantly overexpressed in PD CSF exosomes (Figure 4). With bioinformatics tools the predicted targets of these miRNAs were involved in critical pathways for PD; neurotrophin signaling, mTOR signaling, ubiquitin-mediated proteolysis, dopaminergic synapse, and glutamatergic synapse. To complete the study, authors analyzed exosomal miRNAs in AD too, as we mentioned before in this review article. They conclude that exosomal RNAs could be useful to distinguish accurately between PD and AD (Gui et al., 2015).

With respect to the research of new biomarkers for early diagnosis of PD, Dos Santos et al. (2018) combining an optimized technique of exosomal miRNA isolation with small RNA sequencing, they detected 1,683 exosomal miRNAs in the CSF on 40 early-stage PD patients and 40 well-matched controls. Then, using machine learning approach to find the best miRNA biomarkers for the accurate diagnosis of early-stage PD, they restricted analysis on a panel model of 5 microRNAs, let-7f-5p, miR-27a-3p, miR-125a-5p, miR-151a-3p, and miR-423-5p. Intriguingly, when combining miRNA profiles to protein analysis of the most studied PD related proteins as biomarkers, as DJ-1, UCHL1 and α-synuclein, the robustness of the generated model increased. This work was worth to be mentioned because it is the first study integrating the state-of-the-art microRNA sequencing with protein analysis and complex machine learning approach and obtained potential PD biomarkers in CSF exosomes able to discriminate early PD from healthy controls (Dos Santos et al., 2018). Unfortunately, studies are still scarce and need further investigations and validations although they are promising in the field of biomarker research.

## Concluding Remarks

It is undeniable that last two decades have been characterized by an exponential increase in the number of publications regarding exosomes and their role in the pathogenesis of diseases as well as in the field of clinical biomarker research. Indeed due to their intrinsic ability to transfer biomolecules to other cells and to cross the BBB in both directions, they are becoming an attractive source of potential new biomarkers and/or reservoir of validated ones. The times when exosomes were considered full of junk are long gone. Indeed, new roles and functions for exosomes emerged to the point where these small EVs have been proposed with a double-edged sword role, “Trojan horses” of neurodegeneration or neuroprotective from neurodegeneration. This means that their involvement in neurodegenerative diseases is not totally understood. Some questions remain opened and the most interesting seem to be: (1) are exosomes carrier of disease propagating pathogenic molecules also *in vivo*; (2) can targeting the release of exosomes, or the release of their cargo have an inhibitor effect on the progression of diseases; and (3) can specific protein, or ncRNA signatures isolated from patients be used as biomarker of disease. Further investigations will clarify these aspects as well as the basic research on exosomes improving the comprehension on the role of exosomes in the etiology and progression of these pathologies.

## Author Contributions

MD and MS wrote the article. CF contributed to writing the article and supervised it. BA, MC, ES and DG supervised the final version of the manuscript.

## Conflict of Interest Statement

The authors declare that the research was conducted in the absence of any commercial or financial relationships that could be construed as a potential conflict of interest.

## References

[B1] Alvarez-Erviti L. Seow Y. Yin H. Betts C. Lakhal S. Wood M. J. A. (2011). Delivery of siRNA to the mouse brain by systemic injection of targeted exosomes. Nat. Biotechnol. 29, 341–345. 10.1038/nbt.1807 21423189

[B2] Bellingham S. A. Guo B. B. Coleman B. M. Hill A. F. (2012). Exosomes: vehicles for the transfer of toxic proteins associated with neurodegenerative diseases? Front. Physiol. 3:124. 10.3389/fphys.2012.00124 22563321 PMC3342525

[B3] Benussi L. Ciani M. Tonoli E. Morbin M. Palamara L. Albani D.. (2016). Loss of exosomes in progranulin-associated frontotemporal dementia. Neurobiol. Aging 40, 41–49. 10.1016/j.neurobiolaging.2016.01.001 26973102

[B201] Bhaumik D. Scott G. K. Schokrpur S. Patil C. K. Orjalo A. V. Rodier F.. (2009). MicroRNAs miR-146a/b negatively modulate the senescence-associated inflammatory mediators IL-6 and IL-8. Aging 1, 402–411. 10.18632/aging.100042 20148189 PMC2818025

[B4] Biran A. Zada L. Abou Karam P. Vadai E. Roitman L. Ovadya Y.. (2017). Quantitative identification of senescent cells in aging and disease. Aging Cell 16, 661–671. 10.1111/acel.12592 28455874 PMC5506427

[B202] Brack A. S. Conboy M. J. Roy S. Lee M. Kuo C. J. Keller C.. (2007). Increased Wnt signaling during aging alters muscle stem cell fate and increases fibrosis. Science 317, 807–810. 10.1126/science.1144090 17690295

[B203] Busciglio J. Gabuzda D. H. Matsudaira P. Yankner B. A. (1993). Generation of beta-amyloid in the secretory pathway in neuronal and nonneuronal cells. Proc. Natl. Acad. Sci. U S A 90, 2092–2096. 10.1073/pnas.90.5.2092 8446635 PMC46027

[B6] Caby M. P. Lankar D. Vincendeau-Scherrer C. Raposo G. Bonnerot C. (2005). Exosomal-like vesicles are present in human blood plasma. Int. Immunol. 17, 879–887. 10.1093/intimm/dxh267 15908444

[B7] Campisi J. (2012). Aging, cellular senescence and cancer. Annu. Rev. Physiol. 75, 685–705. 10.1146/annurev-physiol-030212-183653 23140366 PMC4166529

[B8] Campisi J. d’Adda Di Fagagna F. (2007). Cellular senescence: when bad things happen to good cells. Mol. Cell Biol. 8, 729–740. 10.1038/nrm2233 17667954

[B9] Cao X. Y. Lu J. M. Zhao Z. Q. Li M. C. Lu T. An X. S.. (2017). MicroRNA biomarkers of Parkinson’s disease in serum exosome-like microvesicles. Neurosci. Lett. 644, 94–99. 10.1016/j.neulet.2017.02.045 28223160

[B10] Carrettiero D. C. Hernandez I. Neveu P. Papagiannakopoulos T. Kosik K. S. (2009). The cochaperone BAG2 sweeps paired helical filament- insoluble tau from the microtubule. J. Neurosci. 29, 2151–2161. 10.1523/JNEUROSCI 19228967 PMC2768429

[B11] Cheng L. Doecke J. D. Sharples R. A. Villemagne V. L. Fowler C. J. Rembach A.. (2015). Prognostic serum miRNA biomarkers associated with Alzheimer’s disease shows concordance with neuropsychological and neuroimaging assessment. Mol. Psychiatry 20, 1188–1196. 10.1038/mp.2014.127 25349172

[B12] Clavaguera F. Bolmont T. Crowther R. A. Abramowski D. Frank S. Probst A.. (2009). Transmission and spreading of tauopathy in transgenic mouse brain. Nat. Cell Biol. 11, 909–913. 10.1038/ncb1901 19503072 PMC2726961

[B13] Coleman B. M. Hill A. F. (2015). Extracellular vesicles—their role in the packaging and spread of misfolded proteins associated with neurodegenerative diseases. Semin. Cell Dev. Biol. 40, 89–96. 10.1016/j.semcdb.2015.02.007 25704308

[B14] Danzer K. M. Kranich L. R. Ruf W. P. Cagsal-Getkin O. Winslow A. R. Zhu L.. (2012). Exosomal cell-to-cell transmission of α synuclein oligomers. Mol. Neurodegener. 7:42. 10.1186/1750-1326-7-42 22920859 PMC3483256

[B15] Davis C. Dukes A. Drewry M. Helwa I. Johnson M. H. Isales C. M.. (2017). MicroRNA-183–5p increases with age in bone-derived extracellular vesicles, suppresses bone marrow stromal (stem) cell proliferation and induces stem cell senescence. Tissue Eng. Part A 23, 1231–1240. 10.1089/ten.TEA.2016.0525 28363268 PMC5689127

[B16] De Cecco M. Criscione S. W. Peterson A. L. Neretti N. Sedivy J. M. Kreiling J. A. (2013). Transposable elements become active and mobile in the genomes of aging mammalian somatic tissues. Aging 5, 867–883. 10.18632/aging.100621 24323947 PMC3883704

[B17] Ding X. Ma M. Teng J. Teng R. K. F. Zhou S. Yin J.. (2015). Exposure to ALS-FTD-CSF generates TDP-43 aggregates in glioblastoma cells through exosomes and TNTs-like structure. Oncotarget 6, 24178–24191. 10.18632/oncotarget.4680 26172304 PMC4695178

[B18] Dinkins M. B. Dasgupta S. Wang G. Zhu G. Bieberich E. (2014). Exosome reduction *in vivo* is associated with lower amyloid plaque load in the 5XFAD mouse model of Alzheimer’s disease. Neurobiol. Aging 35, 1792–1800. 10.1016/j.neurobiolaging.2014.02.012 24650793 PMC4035236

[B73] Dos Santos M. C. T. Barreto-Sanz M. A. Correia B. R. S. Bell R. Widnall C. Perez L. T.. (2018). miRNA-based signatures in cerebrospinal fluid as potential diagnostic tools for early stage Parkinson’s disease. Oncotarget 9, 17455–17465. 10.18632/oncotarget.24736 29707120 PMC5915128

[B204] Dumic J. Dabelic S. Flögel M. (2006). Galectin-3: an open-ended story. Biochim. Biophys. Acta 1760, 616–635. 10.1016/j.bbagen.2005.12.020 16478649

[B19] Eitan E. Green J. Bodogai M. Mode N. A. Bæk R. Jørgensen M. M.. (2017). Age-related changes in plasma extracellular vesicle characteristics and internalization by leukocytes. Sci. Rep. 7:1342. 10.1038/s41598-017-01386-z 28465537 PMC5430958

[B20] Emmanouilidou E. Melachroinou K. Roumeliotis T. Garbis S. D. Ntzouni M. Margaritis L. H.. (2010). Cell-produced α-synuclein is secreted in a calcium-dependent manner by exosomes and impacts neuronal survival. J. Neurosci. 30, 6838–6851. 10.1523/jneurosci.5699-09.2010 20484626 PMC3842464

[B5] Fauré J. Lachenal G. Court M. Hirrlinger J. Chatellard-Causse C. Blot B.. (2006). Exosomes are released by cultured cortical neurones. Mol. Cell. Neurosci. 31, 642–648. 10.1016/j.mcn.2005.12.003 16446100

[B21] Feiler M. S. Strobel B. Freischmidt A. Helferich A. M. Kappel J. Brewer B. M.. (2015). TDP-43 is intercellularly transmitted across axon terminals. J. Cell Biol. 211, 897–911. 10.1083/jcb.201504057 26598621 PMC4657165

[B22] Fenoglio C. Scarpini E. Galimberti D. (2018). Epigenetic regulatory modifications in genetic and sporadic frontotemporal dementia. Expert Rev. Neurother. 18, 469–475. 10.1080/14737175.2018.1481389 29799291

[B23] Fernando M. R. Jiang C. Krzyzanowski G. D. Ryan W. L. (2017). New evidence that a large proportion of human blood plasma cell-free DNA is localized in exosomes. PLoS One 12:e0183915. 10.1371/journal.pone.0183915 28850588 PMC5574584

[B24] Fiandaca M. S. Kapogiannis D. Mapstone M. Boxer A. Eitan E. Schwartz J. B.. (2015). Identification of preclinical Alzheimer’s disease by a profile of pathogenic proteins in neurally derived blood exosomes: a case-control study. Alzheimers Dement. 11, 600.e1–607.e1. 10.1016/j.jalz.2014.06.008 25130657 PMC4329112

[B25] Franzen C. A. Blackwell R. H. Foreman K. E. Kuo P. C. Flanigan R. C. Gupta G. N. (2015). Urinary exosomes: the potential for biomarker utility, intercellular signaling and therapeutics in urological malignancy. J. Urol. 195, 1331–1339. 10.1016/j.juro.2015.08.115 26714199

[B26] Fraser K. B. Moehle M. S. Daher J. P. L. Webber P. J. Williams J. Y. Stewart C. A.. (2013). LRRK2 secretion in exosomes is regulated by 14–3-3. Hum. Mol. Genet. 22, 4988–5000. 10.1093/hmg/ddt346 23886663 PMC3836478

[B28] Goetzl E. J. Abner E. L. Jicha G. A. Kapogiannis D. Schwartz J. B. (2018). Declining levels of functionally specialized synaptic proteins in plasma neuronal exosomes with progression of Alzheimer’s disease. FASEB J. 32, 888–893. 10.1096/fj.201700731r 29025866 PMC5888398

[B29] Goetzl E. J. Boxer A. Schwartz J. B. Abner E. L. Petersen R. C. Miller B. L.. (2015). Low neural exosomal levels of cellular survival factors in Alzheimer’s disease. Ann. Clin. Transl. Neurol. 2, 769–773. 10.1002/acn3.211 26273689 PMC4531059

[B30] Goetzl E. J. Kapogiannis D. Schwartz J. B. Lobach I. V. Goetzl L. Abner E. L.. (2016). Decreased synaptic proteins in neuronal exosomes of frontotemporal dementia and Alzheimer’s disease. FASEB J. 30, 4141–4148. 10.1096/fj.201600816r 27601437 PMC5102122

[B31] Goldie B. J. Dun M. D. Lin M. Smith N. D. Verrills N. M. Dayas C. V.. (2014). Activity-associated miRNA are packaged in Map1b-enriched exosomes released from depolarized neurons. Nucleic Acids Res. 42, 9195–9208. 10.1093/nar/gku594 25053844 PMC4132720

[B32] Gomes de Andrade G. Reck Cechinel L. Bertoldi K. Galvão F. Valdeci Worm P. Rodrigues Siqueira I. (2018). The aging process alters IL-1β and CD63 levels differently in extracellular vesicles obtained from the plasma and cerebrospinal fluid. Neuroimmunomodulation 25, 18–22. 10.1159/000488943 30021215

[B33] Grey M. Dunning C. J. Gaspar R. Grey C. Brundin P. Sparr E.. (2015). Acceleration of α-synuclein aggregation by exosomes. J. Biol. Chem. 290, 2969–2982. 10.1074/jbc.M114.585703 25425650 PMC4317028

[B206] Guescini M. Genedani S. Stocchi V. Agnati L. F. (2010). Astrocytes and glioblastoma cells release exosomes carrying mtDNA. J. Neural Transm. 117, 1–4. 10.1007/s00702-009-0288-8 19680595

[B34] Gui Y. Liu H. Zhang L. Lv W. Hu X. (2015). Altered microRNA profiles in cerebrospinal fluid exosome in Parkinson disease and Alzheimer disease. Oncotarget 6, 37043–37053. 10.18632/oncotarget.6158 26497684 PMC4741914

[B35] Hébert S. S. Papadopoulou A. S. Smith P. Galas M. C. Planel E. Silahtaroglu A. N.. (2010). Genetic ablation of dicer in adult forebrain neurons results in abnormal tau hyperphosphorylation and neurodegeneration. Hum. Mol. Genet. 19, 3959–3969. 10.1093/hmg/ddq311 20660113

[B207] Hofmann J. W. McBryan T. Adams P. D. Sedivy J. M. (2014). The effects of aging on the expression of Wnt pathway genes in mouse tissues. Age 36:9618. 10.1007/s11357-014-9618-3 24488586 PMC4082588

[B36] Hough K. P. Trevor J. L. Strenkowski J. G. Wang Y. Chacko B. K. Tousif S.. (2018). Exosomal transfer of mitochondria from airway myeloid-derived regulatory cells to T cells. Redox Biol. 18, 54–64. 10.1016/j.redox.2018.06.009 29986209 PMC6031096

[B37] Hu H.-Y. Yu C.-H. Zhang H.-H. Zhang S.-Z. Yu W.-Y. Yang Y.. (2019). Exosomal miR-1229 derived from colorectal cancer cells promotes angiogenesis by targeting HIPK2. Int. J. Biol. Macromol. 132, 470–477. 10.1016/j.ijbiomac.2019.03.221 30936013

[B38] Iguchi Y. Eid L. Parent M. Soucy G. Bareil C. Riku Y.. (2016). Exosome secretion is a key pathway for clearance of pathological TDP-43. Brain 139, 3187–3201. 10.1093/brain/aww237 27679482 PMC5840881

[B39] Ivanov A. Pawlikowski J. Manoharan I. van Tuyn J. Nelson D. M. Rai T. S.. (2013). Lysosome-mediated processing of chromatin in senescence. J. Cell Biol. 202, 129–143. 10.1083/jcb.201212110 23816621 PMC3704985

[B208] Jan A. T. Azam M. Rahman S. Almigeiti A. M. S. Choi D. H. Lee E. J.. (2017). Perspective insights into disease progression, diagnostics and therapeutic approaches in Alzheimer’s disease: a judicious update. Front. Aging Neurosci. 9:356. 10.3389/fnagi.2017.00356 29163138 PMC5671974

[B209] Jan A. T. Rahman S. Khan S. Tasduq S. A. Choi I. (2019). Biology, pathophysiological role, and clinical implications of exosomes: a critical appraisal. Cells 8:E99. 10.3390/cells8020099 30699987 PMC6406279

[B40] Joshi P. Benussi L. Furlan R. Ghidoni R. Verderio C. (2015). Extracellular vesicles in Alzheimer’s disease: friends or foes? focus on Aβ-vesicle interaction. Int. J. Mol. Sci. 16, 4800–4813. 10.3390/ijms16034800 25741766 PMC4394450

[B41] Kapogiannis D. Boxer A. Schwartz J. B. Abner E. L. Biragyn A. Masharani U.. (2015). Dysfunctionally phosphorylated type 1 insulin receptor substrate in neural-derived blood exosomes of preclinical Alzheimer’s disease. FASEB J. 29, 589–596. 10.1096/fj.14-262048 25342129 PMC4314222

[B42] Kenwrick S. (2002). Neural cell recognition molecule L1: relating biological complexity to human disease mutations. Hum. Mol. Genet. 9, 879–886. 10.1093/hmg/9.6.879 10767310

[B43] Kim K. M. Abdelmohsen K. Mustapic M. Kapogiannis D. Gorospe M. (2017). RNA in extracellular vesicles. Wiley Interdiscip. Rev. RNA 8:e1413. 10.1002/wrna.1413 28130830 PMC5474163

[B44] Kokubo H. Saido T. C. Iwata N. Helms J. B. Shinohara R. Yamaguchi H. (2004). Part of membrane-bound Aβ exists in rafts within senile plaques in Tg2576 mouse brain. Neurobiol. Aging 26, 409–418. 10.1016/j.neurobiolaging.2004.04.008 15653169

[B45] Kong S. M. Y. Chan B. K. K. Park J. S. Hill K. J. Aitken J. B. Cottle L.. (2014). Parkinson’s disease-linked human PARK9/ATP13A2 maintains zinc homeostasis and promotes α-Synuclein externalization *via* exosomes. Hum. Mol. Genet. 23, 2816–2833. 10.1093/hmg/ddu099 24603074

[B46] Kosik K. S. (2006). The neuronal microRNA system. Nat. Rev. Neurosci. 7, 911–920. 10.1038/nrn2037 17115073

[B47] Kramer M. L. Schulz-Schaeffer W. J. (2007). Presynaptic α-synuclein aggregates, not lewy bodies, cause neurodegeneration in dementia with lewy bodies. J. Neurosci. 27, 1405–1410. 10.1523/jneurosci.4564-06.2007 17287515 PMC6673583

[B63] Lachenal G. Pernet-Gallay K. Chivet M. Hemming F. J. Belly A. Bodon G.. (2010). Release of exosomes from differentiated neurons and its regulation by synaptic glutamatergic activity. Mol. Cell. Neurosci. 46, 409–418. 10.1016/j.mcn.2010.11.004 21111824

[B48] Lee J. Y. Kim H. S. (2017). Extracellular vesicles in neurodegenerative diseases: a double-edged sword. Tissue Eng. Regen. Med. 14, 667–678. 10.1007/s13770-017-0090-x 30603519 PMC6171665

[B50] Lehmann B. D. Paine M. S. Brooks A. M. McCubrey J. A. Renegar R. H. Wang R.. (2008). Senescence-associated exosome release from human prostate cancer cells. Cancer Res. 68, 7864–7871. 10.1158/0008-5472.can-07-6538 18829542 PMC3845029

[B51] Lim Y. J. Lee S. J. (2017). Are exosomes the vehicle for protein aggregate propagation in neurodegenerative diseases? Acta Neuropathol. Commun. 5:64. 10.1186/s40478-017-0467-z 28851422 PMC5576311

[B52] Liu C. G. Song J. Zhang Y. Q. Wang P. C. (2014). MicroRNA-193b is a regulator of amyloid precursor protein in the blood and cerebrospinal fluid derived exosomal microRNA- 193b is a biomarker of Alzheimer’s disease. Mol. Med. Rep. 10, 2395–2400. 10.3892/mmr.2014.2484 25119742

[B53] Lugli G. Cohen A. M. Bennett D. A. Shah R. C. Fields C. J. Hernandez A. G.. (2015). Plasma exosomal miRNAs in persons with and without Alzheimer disease: altered expression and prospects for biomarkers. PLoS One 10:e0139233. 10.1371/journal.pone.0139233 26426747 PMC4591334

[B210] Ma B. Hottiger M. O. (2016). Crosstalk between Wnt/β-catenin and NF-κB signaling pathway during inflammation. Front. Immunol. 7:378. 10.3389/fimmu.2016.00378 27713747 PMC5031610

[B54] Machida T. Tomofuji T. Ekuni D. Maruyama T. Yoneda T. Kawabata Y.. (2015). MicroRNAs in salivary exosome as potential biomarkers of aging. Int. J. Mol. Sci. 16, 21294–21309. 10.3390/ijms160921294 26370963 PMC4613253

[B55] Mashouri L. Yousefi H. Aref A. R. Ahadi A. M. Molaei F. Alahari S. K. (2019). Exosomes: composition, biogenesis, and mechanisms in cancer metastasis and drug resistance. Mol. Cancer 18:75. 10.1186/s12943-019-0991-5 30940145 PMC6444571

[B56] Molinuevo J. L. Ayton S. Batrla R. Bednar M. M. Bittner T. Cummings J. (2018). Current State of Alzheimer’s Fluid Biomarkers. Berlin: Springer. 10.1007/s00401-018-1932-x PMC6280827 30488277

[B57] Morales R. Duran-Aniotz C. Castilla J. Estrada L. D. Soto C. (2011). *De novo* induction of amyloid-β deposition *in vivo*. Mol. Psychiatry 17, 1347–1353. 10.1038/mp.2011.120 21968933

[B58] Nowak J. S. Michlewski G. (2013). miRNAs in development and pathogenesis of the nervous system: table 1. Biochem. Soc. Trans. 41, 815–820. 10.1042/bst20130044 23863137

[B211] Nusse R. (2005). Wnt signaling in disease and in development. Cell Res. 15, 28–32. 10.1038/sj.cr.7290260 15686623

[B212] Okamoto M. Inoue K. Iwamura H. Terashima K. Soya H. Asashima M.. (2011). Reduction in paracrine Wnt3 factors during aging causes impaired adult neurogenesis. FASEB J. 25, 3570–3582. 10.1096/fj.11-184697 21746862

[B59] Olanow C. W. Brundin P. (2013). Parkinson’s disease and alpha synuclein: is Parkinson’s disease a prion-like disorder? Mov. Disord. 28, 31–40. 10.1002/mds.25373 23390095

[B60] Olivieri F. Albertini M. C. Orciani M. Ceka A. Cricca M. Procopio A. D.. (2015). DNA damage response (DDR) and senescence: shuttled inflamma-miRNAs on the stage of inflamm-aging. Oncotarget 6, 35509–35521. 10.18632/oncotarget.5899 26431329 PMC4742121

[B61] Panagiotou N. Neytchev O. Selman C. Shiels P. (2018). Extracellular vesicles, ageing, and therapeutic interventions. Cells 7:E110. 10.3390/cells7080110 30126173 PMC6115766

[B62] Pegtel D. M. Cosmopoulos K. Thorley-Lawson D. A. van Eijndhoven M. A. J. Hopmans E. S. Lindenberg J. L.. (2010). Functional delivery of viral miRNAs *via* exosomes. Proc. Natl. Acad. Sci. U S A 107, 6328–6333. 10.1073/pnas.0914843107 20304794 PMC2851954

[B213] Perez-Gonzalez R. Gauthier S. A. Kumar A. Levy E. (2012). The exosome secretory pathway transports amyloid precursor protein carboxyl-terminal fragments from the cell into the brain extracellular space. J. Biol. Chem. 287, 43108–43115. 10.1074/jbc.m112.404467 23129776 PMC3522305

[B64] Polanco J. C. Scicluna B. J. Hill A. F. Götz J. (2016). Extracellular vesicles isolated from the brains of rTg4510 mice seed tau protein aggregation in a threshold-dependent manner. J. Biol. Chem. 291, 12445–12466. 10.1074/jbc.m115.709485 27030011 PMC4933440

[B65] Pusic A. D. Kraig R. P. (2014). Youth and environmental enrichment generate serum exosomes containing miR-219 that promote CNS myelination. Glia 62, 284–299. 10.1002/glia.22606 24339157 PMC4096126

[B66] Rademakers R. Hutton M. (2007). The genetics of frontotemporal lobar degeneration. Curr. Neurol. Neurosci. Rep. 7, 434–442. 10.1007/s11910-007-0067-6 17764635

[B67] Rajendran L. Bali J. Barr M. M. Court F. A. Kramer-Albers E.-M. Picou F.. (2014). Emerging roles of extracellular vesicles in the nervous system. J. Neurosci. 34, 15482–15489. 10.1523/JNEUROSCI.3258-14.2014 25392515 PMC4228143

[B68] Rajendran L. Honsho M. Zahn T. R. Keller P. Geiger K. D. Verkade P.. (2006). Alzheimer’s disease β-amyloid peptides are released in association with exosomes. Proc. Natl. Acad. Sci. U S A 103, 11172–11177. 10.1073/pnas.0603838103 16837572 PMC1544060

[B69] Rashed M. H. Bayraktar E. Helal G. K. Abd-Ellah M. F. Amero P. Chavez-Reyes A.. (2017). Exosomes: from garbage bins to promising therapeutic targets. Int. J. Mol. Sci. 18:E538. 10.3390/ijms18030538 28257101 PMC5372554

[B70] Ridder K. Keller S. Dams M. Rupp A. K. Schlaudraff J. Del Turco D.. (2014). Extracellular vesicle-mediated transfer of genetic information between the hematopoietic system and the brain in response to inflammation. PLoS Biol. 12:e1001874. 10.1371/journal.pbio.1001874 24893313 PMC4043485

[B71] Salvioli S. Monti D. Lanzarini C. Conte M. Pirazzini C. Bacalini M. G.. (2013). Immune system, cell senescence, aging and longevity—inflamm-aging reappraised. Curr. Pharm. Des. 19, 1675–1679. 10.2174/138161213805219531 23589904

[B72] Saman S. Lee N. C. Y. Hall G. F. Saman S. Visnick Y. Jackson B.. (2012). Exosome-associated tau is secreted in tauopathy models and is selectively phosphorylated in cerebrospinal fluid in early Alzheimer disease. J. Biol. Chem. 287, 3842–3849. 10.1074/jbc.m111.277061 22057275 PMC3281682

[B74] Schneider R. McKeever P. Kim T. Graff C. van Swieten J. C. Karydas A.. (2018). Downregulation of exosomal miR-204–5p and miR-632 as a biomarker for FTD: a GENFI study. J. Neurol. Neurosurg. Psychiatry 89, 851–858. 10.1136/jnnp-2017-317492 29434051 PMC6045452

[B75] Sharples R. Vella L. J. Nisbet R. M. Naylor R. Perez K. Barnham K. J.. (2008). Inhibition of γ-secretase causes increased secretion of amyloid precursor protein C-terminal fragments in association with exosomes. FASEB J. 22, 1469–1478. 10.1096/fj.07-9357com 18171695

[B76] Shi M. Kovac A. Korff A. Cook T. J. Ginghina C. Kristin M.. (2017). CNS tau efflux *via* exosomes is likely increased in Parkinson’s disease but not in Alzheimer’s disease. Alzheimers Dement. 12, 1125–1131. 10.1016/j.jalz.2016.04.003 27234211 PMC5107127

[B77] Shi M. Liu C. Cook T. J. Bullock K. M. Zhao Y. Ginghina C.. (2014). Plasma exosomal α-synuclein is likely CNS-derived and increased in Parkinson’s disease. Acta Neuropathol. 128, 639–650. 10.1007/s00401-014-1314-y 24997849 PMC4201967

[B78] Shi M. Sheng L. Stewart T. Zabetian C. P. Zhang J. (2019). New windows into the brain: central nervous system-derived extracellular vesicles in blood. Prog. Neurobiol. 175, 96–106. 10.1016/j.pneurobio.2019.01.005 30685501 PMC6546433

[B79] Shiels P. G. Stenvinkel P. Kooman J. P. McGuinness D. (2017). Circulating markers of ageing and allostatic load: a slow train coming. Pract. Lab. Med. 7, 49–54. 10.1016/j.plabm.2016.04.002 28856219 PMC5574864

[B80] Simón D. García-García E. Gómez-Ramos A. Falcón-Pérez J. M. Díaz-Hernández M. Hernández F.. (2012). Tau overexpression results in its secretion *via* membrane vesicles. Neurodegener. Dis. 10, 73–75. 10.1159/000334915 22269430

[B81] Simons M. Raposo G. (2009). Exosomes—vesicular carriers for intercellular communication. Curr. Opin. Cell Biol. 21, 575–581. 10.1016/j.ceb.2009.03.007 19442504

[B82] Snowden J. Neary D. Mann D. (2007). Frontotemporal lobar degeneration: clinical and pathological relationships. Acta Neuropathol. 114, 31–38. 10.1007/s00401-007-0236-3 17569065

[B83] Soria F. N. Pampliega O. Bourdenx M. Meissner W. G. Bezard E. Dehay B. (2017). Exosomes, an unmasked culprit in neurodegenerative diseases. Front. Neurosci. 11:26. 10.3389/fnins.2017.00026 28197068 PMC5281572

[B84] Spillantini M. G. Goedert M. (2018). Neurodegeneration and the ordered assembly of α-synuclein. Cell Tissue Res. 373, 137–148. 10.1007/s00441-017-2706-9 29119326 PMC6015613

[B85] Sprott R. L. (2010). Biomarkers of aging and disease: introduction and definitions. Exp. Gerontol. 45, 2–4. 10.1016/j.exger.2009.07.008 19651201

[B86] Stuendl A. Kunadt M. Kruse N. Bartels C. Moebius W. Danzer K. M.. (2016). Induction of α-synuclein aggregate formation by CSF exosomes from patients with Parkinson’s disease and dementia with Lewy bodies. Brain 139, 481–494. 10.1093/brain/awv346 26647156 PMC4805087

[B87] Takasugi M. (2018). Emerging roles of extracellular vesicles in cellular senescence and aging. Aging Cell 17:2. 10.1111/acel.12734 29392820 PMC5847882

[B88] Takasugi M. Okada R. Takahashi A. Virya Chen D. Watanabe S. Hara E. (2017). Small extracellular vesicles secreted from senescent cells promote cancer cell proliferation through EphA2. Nat. Commun. 8:15729. 10.1038/ncomms15728 28585531 PMC5467215

[B214] Takahashi R. H. Milner T. A. Li F. Nam E. E. Edgar M. A. Yamaguchi H.. (2002). Intraneuronal Alzheimer Aβ42 accumulates in multivesicular bodies and is associated with synaptic pathology. Am. J. Pathol. 161, 1869–1879. 10.1016/s0002-9440(10)64463-x 12414533 PMC1850783

[B89] Thomas B. Beal M. F. (2007). Parkinson’s disease. Hum. Mol. Genet. 16, R183–R194. 10.1093/hmg/ddm159 17911161

[B90] Thompson A. G. Gray E. Heman-Ackah S. M. Mäger I. Talbot K. El Andaloussi S.. (2016). Extracellular vesicles in neurodegenerative disease-pathogenesis to biomarkers. Nat. Rev. Neurol. 12, 346–357. 10.1038/nrneurol.2016.68 27174238

[B91] Tsunemi T. Hamada K. Krainc D. (2014). ATP13A2/PARK9 regulates secretion of exosomes and α-synuclein. J. Neurosci. 34, 15281–15287. 10.1523/jneurosci.1629-14.2014 25392495 PMC4228131

[B92] Urbanelli L. Buratta S. Sagini K. Tancini B. Emiliani C. (2016). Extracellular vesicles as new players in cellular senescence. Int. J. Mol. Sci. 17:E1408. 10.3390/ijms17091408 27571072 PMC5037688

[B93] Valadi H. Ekström K. Bossios A. Sjöstrand M. Lee J. J. Lötvall J. O. (2007). Exosome-mediated transfer of mRNAs and microRNAs is a novel mechanism of genetic exchange between cells. Nat. Cell Biol. 9, 654–659. 10.1038/ncb1596 17486113

[B94] van Balkom B. W. M. de Jong O. G. Smits M. Brummelman J. den Ouden K. de Bree P. M.. (2013). Endothelial cells require miR-214 to secrete exosomes that suppress senescence and induce angiogenesis in human and mouse endothelial cells. Blood 121, 3997–4006. 10.1182/blood-2013-02-478925 23532734

[B95] Vidal M. Sainte-Marie J. Philippot J. R. Bienvenue A. (1989). Asymmetric distribution of phospholipids in the membrane of vesicles released during *in vitro* maturation of guinea pig reticulocytes: evidence precluding a role for “aminophospholipid translocase”. J. Cell. Physiol. 140, 455–462. 10.1002/jcp.1041400308 2777884

[B96] Wei H. Xu Y. Xu W. Zhou Q. Chen Q. Yang M.. (2018). Serum exosomal miR-223 serves as a potential diagnostic and prognostic biomarker for dementia. Neuroscience 379, 167–176. 10.1016/j.neuroscience.2018.03.016 29559383

[B97] Weilner S. Keider V. Winter M. Harreither E. Salzer B. Weiss F.. (2016). Vesicular Galectin-3 levels decrease with donor age and contribute to the reduced osteo-inductive potential of human plasma derived extracellular vesicles. Aging 8, 16–30. 10.18632/aging.100865 26752347 PMC4761711

[B98] Weilner S. Schraml E. Redl H. Grillari-Voglauer R. Grillari J. (2013). Secretion of microvesicular miRNAs in cellular andorganismal aging. Exp. Gerontol. 48, 626–633. 10.1016/j.exger.2012.11.017 23283304 PMC3695566

[B99] Weiner-Gorzel K. Dempsey E. Milewska M. McGoldrick A. Toh V. Walsh A.. (2015). Overexpression of the microRNA miR-433 promotes resistance to paclitaxel through the induction of cellular senescence in ovarian cancer cells. Cancer Med. 4, 745–758. 10.1002/cam4.409 25684390 PMC4430267

[B100] Westergard T. Jensen B. K. Wen X. Cai J. Kropf E. Iacovitti L.. (2016). Cell-to-cell transmission of dipeptide repeat proteins linked to C9orf72 -ALS/FTD. Cell Rep. 17, 645–652. 10.1016/j.celrep.2016.09.032 27732842 PMC5078984

[B101] Willms E. Johansson H. J. Mäger I. Lee Y. Blomberg K. E. M. Sadik M.. (2016). Cells release subpopulations of exosomes with distinct molecular and biological properties. Sci. Rep. 6:22519. 10.1038/srep22519 26931825 PMC4773763

[B102] Winston C. N. Goetzl E. J. Akers J. C. Carter B. S. Rockenstein E. M. Galasko D.. (2016). Prediction of conversion from mild cognitive impairment to dementia with neuronally derived blood exosome protein profile. Alzheimers Dement. 3, 63–72. 10.1016/j.dadm.2016.04.001 27408937 PMC4925777

[B103] Xu Y. Sun Z. (2015). Molecular basis of Klotho: from gene to function in aging. Endocr. Rev. 36, 174–193. 10.1210/er.2013-1079 25695404 PMC4399270

[B27] Yagi Y. Ohkubo T. Kawaji H. Machida A. Miyata H. Goda S.. (2016). Next-generation sequencing-based small RNA profiling of cerebrospinal fluid exosomes. Neurosci. Lett. 636, 48–57. 10.1016/j.neulet.2016.10.042 27780738

[B104] Yamakuchi M. Lowenstein C. J. (2009). MiR-34, SIRT1 and p53: the feedback loop. Cell Cycle 8, 712–715. 10.4161/cc.8.5.7753 19221490

[B105] Yang T. T. Liu C. G. Gao S. C. Zhang Y. Wang P. C. (2018). The serum exosome derived MicroRNA-135a, -193b, and -384 were potential Alzheimer’s disease biomarkers. Biomed. Environ. Sci. 31, 87–96. 10.3967/bes2018.011 29606187

[B106] Yuyama K. Sun H. Usuki S. Sakai S. Hanamatsu H. Mioka T.. (2015). A potential function for neuronal exosomes: sequestering intracerebral amyloid-β peptide. FEBS Lett. 589, 84–88. 10.1016/j.febslet.2014.11.027 25436414

[B107] Zhang G. Yang P. (2018). A novel cell-cell communication mechanism in the nervous system: exosomes. J. Neurosci. Res. 96, 45–52. 10.1002/jnr.24113 28718905

[B215] Zhang Y. Pizzute T. Pei M. (2014). A review of crosstalk between MAPK and Wnt signals and its impact on cartilage regeneration. Cell Tissue Res. 358, 633–649. 10.1007/s00441-014-2010-x 25312291 PMC4234693

